# A decade of adverse drug events in Portuguese hospitals: space-time clustering and spatial variation in temporal trends

**DOI:** 10.1186/s40360-017-0140-y

**Published:** 2017-05-10

**Authors:** Gianina Scripcaru, Ceu Mateus, Carla Nunes

**Affiliations:** 1 Escola Nacional de Saúde Pública, Universidade NOVA de Lisboa, Av Padre Cruz, 1600-560 Lisbon, Portugal; 2 AMGEN Biofarmaceutica, Lisbon, Portugal; 3 Health Economics Group Division of Health Research, Lancaster University, Lancaster, UK; 4 Centro de Investigação em Saúde Pública, Universidade NOVA de Lisboa, Lisbon, Portugal

**Keywords:** Adverse drug events, Hospitals, Space-time, Epidemiology

## Abstract

**Background:**

The aim of this study is to identify the distribution by municipalities of adverse drug events (ADE) in Portugal, including adverse drug reactions (ADR) and accidental poisoning by drugs (AP), on municipality/years ADE rate clustering. Also we identify areas with different trends in time.

**Methods:**

We used a national dataset of public hospital discharges in Continental Portugal from 2004 to 2013. Events were identified based on codes: from E930 to E949.9 (ADR) and from E850 to E858.9 (AP). Space-time clustering and spatial variation in temporal trends methods were applied in three different time-periods: globally, by year and grouped in 2 classes (periods of 5 years).

**Results:**

A total of 9,320,076 patients were discharged within this period, with 133,688 patients (1.46%) having at least one ADE, 4% of them related with AP. Critical space-time identified clusters (p < 0.001) were the municipalities from Lisbon metropolitan area and Centro region area. The global rate increased at a 7.8% mean annual percentage change, with high space-time heterogeneity and variation in time trends clusters (p < 0.001). For whole period, 2004–2013, all clusters presented increasing trends. However when analyzed by period of 5 years we identified two clusters with decreasing trends in time in 2004–2008.

**Conclusion:**

The impact of ADE is huge, with widely variations within country and in time, and represents an increasing challenge. Future research using individual and contextual risk factors are urgently needed to understand this spatiotemporal variability in order to promote local tailored and updated actions of prevention.

## Background

The use of the drugs, in addition to the benefit brought, may include some risks, is that at some point the patient may suffer an iatrogenic process, which can cause injuries with different levels of severity, potentially requiring patient hospitalization [1].

In the last decade organizational structures of pharmacovigilance have been continuously improved, with a closer supervision of the drugs over a long period of time including after the approval of commercialization. Pharmacovigilance is a key element of effective activity with the drugs, the medical practice and public health programs [2].

Studies have estimated a varied percentage of incidences of adverse events related with the drugs leading to hospitalization ranges from 2.4% to 6.5% of all hospitalizations in Western European countries [3–6]. Regarding the elderly, this percentage is higher, ranging between 3.4% and 16.6% [3, 4]. The percentage of patients with one or more adverse drug events during hospitalization varies between 1.8% and 19.2%, resulting in increased hospitalization time and thus the costs [7, 8]. In Lagnaoui et al., the incidence rate for in-hospital adverse drug reactions was 10.1 per 1000 patient-days [9]. Geographic epidemiology studies can help to detect regions with rising incidences of adverse drug events and differences in drug utilization [10].

In literature review of Patel and Peter in 2002 regarding the visits to the emergency department, it was estimated that 28% of these were due to adverse drug events, of which 24% required hospitalization, and 70–80% were avoidable adverse events [11, 12].

Geographical information systems and spatial analysis provide important tools for disease control, where the place can be considered as proxy for the interaction between genetic factors, lifestyle and environment [13, 14]. Spatial autocorrelation statistics provide useful information about the spatial arrangement of data in a map and the correlation or dependency rates for geographically close areas with application in several sciences such as criminology, ecology, public health [15–18]. Spatial scan statistics [15] is one of the most used methods, identifying potential clusters by drawing circles of different sizes over the area of study and compare the risk of disease inside and outside of each circle [18]. Advances in methodology of spatial epidemiology create opportunities for researcher to improve reporting of disease at national or regional scale, even in small areas, although this studies are susceptible to confusing results related with small numbers [19]. Two methodological methods were applied in this study, space–time clustering and spatial variation in temporal trends, enabling the identification of areas where major problems and areas with temporal trends different from the rest of the country. The use of these two methods is a very powerful tool to improve knowledge in public health.

The study objectives are to identify spatiotemporal municipality-years incidence rate high clusters of adverse drug events (ADE) based on the hospital discharges dataset in Portugal from 2004 to 2013 and find spatial clusters of incidence with different trends in the same period. This study aims to contribute to public health management focused on adverse drug events and as we know, is the first nationwide study using this methodology.

## Methods

Using the Portuguese hospital inpatient discharges dataset from 2004 to 2013 a retrospective observational nationwide study was using the International Classification of Diseases (ICD-9). In the analysis were included patients with ADE by geographical area being the geographical unit “municipality” - in mainland Portugal (*n* = 278).

The dataset has patients’ anonymized information and the variables used were: year, sex, age, length of stay, discharge status (home, transferred, dead, and so on), hospital’s region (of five Regional Health Administrations – RHA) and external causes (E codes). All day episodes were excluded from the analyses. ADE were defined as injuries resulting from the use of prescription and over-the-counter medications for medical intervention, which includes adverse drug reactions and medication error and excludes administration of the wrong medication, intentional overdoses or use of illicit substances, in agreement with the literature [20–24].

Based on literature, the following E codes were used from ICD-9: E930 to E949.9 of ADR and E codes from E850 to E858.9 (specifically for AP) [24–26].

The unit of analysis is annual rate of adverse drug events per 10 000 hospitalizations between 2004 and 2013. Patient admitted multiple times in hospital are accounted for each time separately. For the rate’s numerator were considered annual discharges with adverse drug events by patients’ municipalities. The denominator were taken all annual discharges by year in ten-year’ time period.

As a first approach a descriptive analysis was performed, followed by two clustering methods. First we use the classical space-time clustering to identify high incidence areas, followed by the analysis of the spatial variation in temporal trends. For this, a retrospective space-time analysis using a Poisson distribution for identification of the elevated disease risk within a cluster, using SaTScan Software [27], was done. Cluster analysis can detect and identify geographic areas that have significant differences in risk, regardless of their size. SaTScan software, based in Kulldorff’s statistical methodology, uses the spatial scan and is routinely used in public health [17, 27–29].

A first clustering analysis was conducted for the whole period from 2004 up to 2013. After, based on the obtained results, this period was split in two smaller periods, 2004–2008 and 2009–2013, to evaluate the consistency of clusters found and respective trends. Data were analyzed using a statistical significance level of 5%.

The results were mapped using the Epi Info software [30].

## Results

Table 1 illustrates the distribution of patient discharges (all, ADE and subgroups), globally and by year. The analysis shows a slight increase in the number of all ADE in general, with an increase in ADR since 2005, although the AP are more stable in evolution in the period studied. An increase of 70% for ADE was observed during the period, with 11,007 events in 2004 and reaching 18,750 in 2013.

**Table 1:** Distribution of ADR & AP by year and globally

Year	2004	2005	2006	2007	2008	2009	2010	2011	2012	2013	Total
ADR *N* (%)	10 442 (94.9%)	9 444 (94.5%)	10 911 (95.2%)	11 217 (95.7%)	11 271 (95.7%)	12 404 (96.3%)	13 773 (96.1%)	14 320 (96%)	16 560 (98.2%)	18 262 (97.4%)	128 604 (96.2%)
AP *N* (%)	565 (5.1%)	552 (5.5%)	553 (4.8%)	502 (4.3%)	501 (4.3%)	477 (3.7%)	552 (3.8%)	590 (3.9%)	304 (1.8%)	488 (2.6%)	5 084 (3.8%)
Total ADE *N* (%)	11 007 (100%)	9 996 (100%)	11 464 (100%)	11 719 (100%)	11 772 (100%)	12 881 (100%)	14 325 (100%)	14 910 (100%)	16 864 (100%)	18 750 (100%)	133 688 (100%)
% ADE in all discharges	1.1%	1.1%	1.3%	1.3%	1.3%	1.4%	1.5%	1.7%	1.9%	2.1%	1.5%

Descriptive statistics are presented in Table 2, by year and globally, and also identifying the municipalities with highest ADE rates.

**Table 2:** Description of ADE rates in the 278 municipalities of continental Portugal, globally and yearly

Year	Maximum (Municipality, No. of cases)	Minimum (No. of Municitality, %)	Mean	Median	S.D.
2004	227.9 (Lisboa,1402)	0 (4; 1.4)	92.9	89.1	52.1
2005	285.7 (Mertola, 2)^a^	0 (6; 2.2)	95.4	87.4	56.9
2006	355.8 (Lagoa, 10)	0 (10; 3.5)	106.9	99.5	65.3
2007	735.2 (Castro Verde, 5)^a^	0 (13; 4.6)	108.5	101.1	88.7
2008	510.2 (Vidigueira, 5)^a^	0 (5; 1.8)	111.2	104.4	67.7
2009	592.5 (Ferreira do Alentejo, 8)^a^	0 (5; 1.8)	131.3	122	70.9
2010	349.1 (Sardoal, 14)^b^	9.2 (1; 0.3)	136.5	130.5	60.4
2011	353.9 (Penedono, 8)	18.8 (1; 0.3)	147.1	145.3	66.3
2012	429.7 (Vouzela, 41)^b^	0 (1; 0.3)	168.5	162.1	66.1
2013	501.6 (Penalva do Castelo, 45)^b^	0 (1; 0.3)	209	198.2	83.6
GLOBAL	268.2 (Oeiras, 3791)	31.1 (1; 0.3)	127.5	126.2	42.9

^a^Municipalities from Alentejo Region; ^b^Municipalities from Centro Region

The municipality with the highest ADE rate changed every year. In most of the years (8 out of 10) the minimum rate was zero, with a proportion of municipalities with rate zero between 0.3 and 4.6%. Moreover the higher rates of ADE are concentrated in two country regions, Alentejo (58 municipalities) and Centro (78 municipalities), being these two profiles associated with small discharges numbers.

**Fig. 1:**
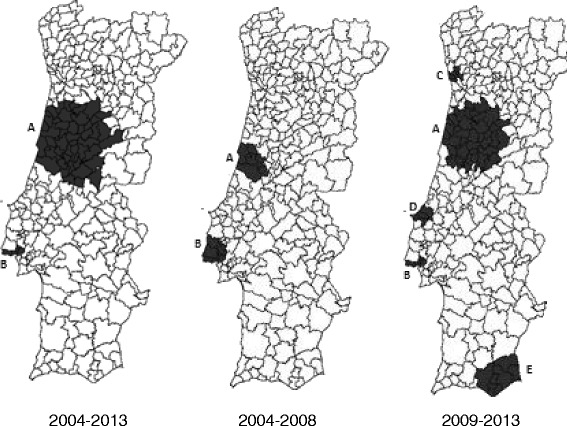
Space-time clusters of higher rate of ADE cases in three periods

Figure 1 presents the space-time clusters of higher rates of ADE in Portugal, in three periods: 2004–2013, 2004–2008 and 2009–2013.

Table 3 presents the characteristics of critical areas (identified clusters), considering the overall period (2004–2013) and the two half periods (2004–2008; 2009–2013). This split was based on 2004–2013 results.

**Table 3:** Space-time clusters with higher rate of ADE in three time periods (2004–2013, 2004–2008 and 2099–2013) (p < 0.001)

Time period	Clusters	No. Of municipalities	Time frame	Observed/expected ratio
2004–2013	A	48	2010–2013	1.36
	B	5	2009–2013	1.75
2004–2008	A	8	2007–2008	1.43
	B	6	2006–2007	1.75
2009–2013	A	39	2012–2013	1.33
	B	5	2012–2013	1.61
	C	5	2012–2013	1.62
	D	4	2012–2013	1.60
	E	8	2012–2013	1.58

**Fig. 2:**
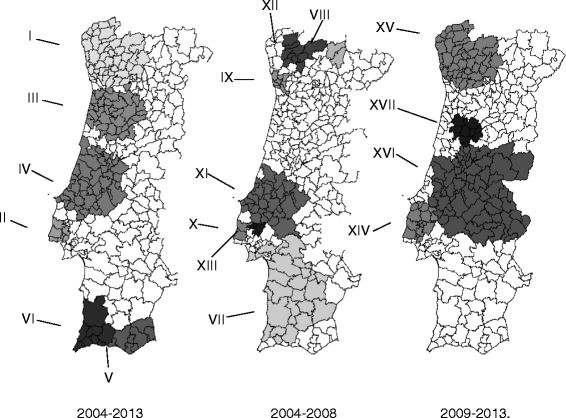
Spatial variation in temporal trends of ADE incidence rate in three periods

Table 4 shows the result for the spatial variation in temporal trends of ADE rates for the three time periods being studied (2004–2013; 2004–2008; 2009–2013). Additionally Figs. 2 present the incidence rate trends and spatial distribution of the identified clusters for the same three studied time-periods.

**Table 4:** Space clusters of temporal trends of ADE incidence rate found in 2004–2013, 2004–2008 and 2009–2013. Positive and negative percentages correspond to increasing or decreasing trends, respectively

Time-period a-b global trend (%)	Clusters (*p* < 0,05)	No. of cases in a-b (no. of municipalities)	Trends (%)	ADE rate x 10¯^4^ in period a	ADE rate x 10¯^4^ in period b
2004–2013	I	16403 (44)	**14,8**	65.8	204.6
(7,83%)	II	36146 (8)	3,8	184.6	253.7
	III	12883 (38)	**11.4**	91.3	202.5
	IV	13528 (40)	4.8	117.3	177.9
	V	3841 (9)	**12.4**	103.4	287.5
	VI	1126 (8)	1.4	79.8	142.1
2004–2008	VII	2997 (26)	**17.1**	95.8	165.1
(3,70%)	VIII	335 (2)	**40.1**	46.1	164.1
	IX	5135 (8)	−2.9	122.1	115.5
	X	2699 (3)	**12.9**	108.2	158
	XI	4216 (30)	−2.2	114.7	103.2
	XII	1157 (10)	**15.9**	62.5	101.4
	XIII	584 (2)	**19.6**	87	150.7
2009–2013	XIV	17598 (15)	4.5	190.7	226.8
(11.20%)	XV	12837 (44)	**19.5**	103.4	204.6
	XVI	7651 (62)	5.9	127.5	169.8
	XVII	1692 (10)	**21.6**	109.4	266.5

Values in bold identified areas where trends were higher than global trends (for each a-b period)

## Discussion

To our knowledge this is the first space-time study aiming to understand the phenomena of adverse drug events at national level in Portugal. This analysis contributes with two important results: 1) the variability in space and time within the country and 2) increasing rate of ADE for the whole time period with all significant clusters increasing above the national mean.

In 2004–2013 period, we have identified an increase proportion of ADE in all discharges (1,1% in 2004 and 2,1% in 2013). Also in this period a slight increase in the number of overall hospitalizations was observed. Many of the side effects are associated with specific factors related with the patient and/or with the drug. The evolution of the treatments alternatives being used (with more and stronger drugs available on the market) and increase in average life expectancy can be considered as principal reasons for ADE. Other factors that contribute to adverse reactions are related with the accuracy of the prescribing, dispensing and administration of the drugs [31]. Risk factors related with patients include older age, female sex, drugs in therapy and the number of associated comorbidities [32–34]. Determining the incidence of adverse drug reactions may be difficult, because frequently drugs are not recognized as the cause of symptoms or diseases [35]. Not all studies accurately disentangle ADE leading to hospitalization and hospital-acquired ADE what becomes a confounder in the results obtained.

When space-time clustering was applied to the whole period 2004–2013, the municipalities from Lisbon metropolitan area (clusters 1) were identified as critical in the 2009–2013 period and Centro region area (cluster 2) was identified as critical in time frame 2010–2013. Using the same analysis for the two sub-periods, with a cut-off point in 2008/2009, similar critical areas were identified in the 2004–2008 period for the years 2006–2007 in the Lisbon metropolitan area (cluster 3) and in years 2007–2008 for the Centro Region (cluster 4). For the second period (2009–2013), we further identified three clusters besides the ones already known. These three clusters are located in the Oporto metropolitan area, Algarve Region and Centro Region. All the clusters in this period are considered as critical for the years 2012 and 2013.

Previous studies have shown the rate of ADR in hospital inpatients ranging from 0.8% to 26.1% [7–9, 36, 37]. Studies using administrative datasets have shown rates of ADR between 0.8% and 1.8% in Europe [26, 37, 38]. The differences in the results observed can be due to differences in the accuracy of data reporting in the datasets used or to the definition of the ADR.

A large number of studies points towards an increasing trend of ADR over time [38–40]. In our study the spatial variation in temporal trends of ADE the mean annual percentage change in the rate was 7.8% for the period 2004–2013, 3.7% for 2004–2008 and 11.2% for 2009–2013. During 2004–2013 six significant clusters were identified and all of them present increasing trends for the ADE’ rate. Clusters II, IV and VI showed a slower increase in the mean annual trend (3.8%, 4.8% and 1.4%) when compared against the country global mean annual trend (7.8%). The mean annual trend for the remaining clusters increased faster than the country mean.

In the period 2004–2008 seven clusters with significant variations were identified. Only two clusters with decreasing trends were found, clusters IX and XI with rates of −2.9% and −2.2%, respectively. In this period cluster VIII showed an accelerated increase with a rate reaching 40.1%, with an increase in the rate of ADE from 46 cases to 164 cases/10 000 inpatients.

In the period 2009–2013 four clusters with significant increases were identified. Clusters XIV and XVI showed a slower increase in their trend when compared with the country mean. In this period Cluster XVII showed an increase in the mean percentage rate (21.6%) that was twice the observed in the country mean.

The mean annual change in rate for the period 2004 to 2013 was 7.8%, although in 2009–2013 an accelerate increase in rate was detected. Globally all clusters present increasing trends, however cluster VI, the smaller one in this period including eight municipalities with 10 112 discharges, shows an irregular trend in 2009. A very accelerate trend was identified for cluster V, belonging to the South region of the country, that was not identified as critical the half periods.

Decreasing trends are only observable in the period 2004–2008, with cluster IX including Oporto metropolitan area and cluster XI placed in region Centro and Alentejo. Comparing with the increasing rate in this period, reaching in cluster VIII an annual increase percentage of 40.1%, the decreasing rate are very small with a percentage rate only −2.9% in cluster IX and −2.2% in cluster XI. Clusters with high increasing trends comprised a small number of municipalities. Such clusters should be analyzed more closely, as their trend may be explained by the variation of small numbers or administrative issues. We must also mention that clusters with decreasing trends (IX and XI) are bigger, as per the number of patients discharged, when compared with the clusters with increasing trends (VII, VIII, X, XII and XIII) in same period. Critical areas, identified through space-time clustering were partially consistent with the municipalities with the maximum rate obtained in the descriptive approach. Space-time clustering analysis is preferred for identified critical areas, even in small numbers although a careful interpretation of this results and future studies must be considered [41].

The number of ADE has increased in the last decade in Portugal, with the subgroup of AP being more stable in time than ADR. High variability across country was observed and the method applied to this study, a combination between space-time clustering and spatial variation in temporal trends, allow the identification of the areas with high risk and with temporal trend different from the rest of the country.

The increase in the frequency of ADE was expected considering several factors that can contribute for ADE (new drugs, population ageing, multiple drugs in therapy, comorbidities etc.) but the variations must be understand [32–34, 42]. Results are consistent with other studies using administrative hospital datasets which report increasing trends of ADE related hospitalizations in England, Germany and Sweden. Current efforts worldwide are now being developed to reduce morbidity and mortality related to drugs and the application of this methodology provides better knowledge to set geographic priorities within the country pharmacovigilance system.

In this analysis we assumed homogeneity in the codification and in the notification rate in space and time. If this situation does not occur biases can affect the study results.

## Conclusion

The mean annual percentage change in ADE rate in the period 2004–2013 was 7.8%, with all clusters in an increase trend. In the first half period 2004–2008, the increase was slighter lower (3.7%) but in the period 2009–2013 we observed an accelerate increase reaching 11.2%. Identified high incidence areas weren’t overlap with trends results, but joint analysis of these results raises very promising prospects for the design of future interventions to control this challenging problem. The impact of ADE is huge, with widely variations within country, and presents an increasing pattern. Future research using individual (comorbidities, concomitant medication, life style or genetic predisposition) and contextual risk factors (regional variability related with health services) can be helpful to explain this spatiotemporal variability in order to promote local tailored and updated actions of prevention.

## Abbreviations

ADE: Adverse drug events
ADR: Adverse drug reactions
AP: Accidental poisoning by drugs
